# Mir‐29b promotes human aortic valve interstitial cell calcification via inhibiting TGF‐β3 through activation of wnt3/β‐catenin/Smad3 signaling

**DOI:** 10.1002/jcb.26545

**Published:** 2018-03-25

**Authors:** Ming Fang, Cheng‐Guang Wang, Changzhu Zheng, Jun Luo, Shiqiang Hou, Kangyong Liu, Xinming Li

**Affiliations:** ^1^ Department of Cardiology Hainan General Hospital Haikou Hainan P.R. China; ^2^ Department of Cardiology Shanghai Zhoupu Hospital Shanghai P.R. China; ^3^ Laboratory of System Biology Shanghai Advanced Research Institute Chinese Academy of Sciences Shanghai P.R. China; ^4^ Department of Neurology Shanghai Zhoupu Hospital Shanghai P.R. China

**Keywords:** calcific aortic valve disease, miR‐29b, Runx2/Smad3/TGF‐β3 signaling, valvular calcification, wnt/β‐catenin signaling

## Abstract

Herein, we hypothesized that pro‐osteogenic MicroRNAs (miRs) could play functional roles in the calcification of the aortic valve and aimed to explore the functional role of miR‐29b in the osteoblastic differentiation of human aortic valve interstitial cells (hAVICs) and the underlying molecular mechanism. Osteoblastic differentiation of hAVICs isolated from human calcific aortic valve leaflets obtained intraoperatively was induced with an osteogenic medium. Alizarin red S staining was used to evaluate calcium deposition. The protein levels of osteogenic markers and other proteins were evaluated using western blotting and/or immunofluorescence while qRT‐PCR was applied for miR and mRNA determination. Bioinformatics and luciferase reporter assay were used to identify the possible interaction between miR‐29b and TGF‐β3. Calcium deposition and the number of calcification nodules were pointedly and progressively increased in hAVICs during osteogenic differentiation. The levels of osteogenic and calcification markers were equally increased, thus confirming the mineralization of hAVICs. The expression of miR‐29b was significantly increased during osteoblastic differentiation. Furthermore, the osteoblastic differentiation of hAVICs was significantly inhibited by the miR‐29b inhibition. TGF‐β3 was markedly downregulated while Smad3, Runx2, wnt3, and β‐catenin were significantly upregulated during osteogenic induction at both the mRNA and protein levels. These effects were systematically induced by miR‐29b overexpression while the inhibition of miR‐29b showed the inverse trends. Moreover, TGF‐β3 was a direct target of miR‐29b. Inhibition of miR‐29b hinders valvular calcification through the upregulation of the TGF‐β3 via inhibition of wnt/β‐catenin and RUNX2/Smad3 signaling pathways.

## 1 INTRODUCTION

Valvular calcification is a common pathological process which is observed extensively in patients with renal disease, type II diabetes, calcific aortic valve diseases (calcific aortic valve disease, CAVD) including active aortic valve calcification and aortic stenosis.^1^, ^2^, ^3^, ^4^, ^5^, ^6^, ^7^ Although the clinical significance of valvular calcification is well‐recognized, the mechanisms involved are still not clear.

MicroRNAs (miRs) have been considered as a nucleus research topic in recent years. They are a group of non‐coding single‐stranded cellular RNAs composed of 18–25 nucleotides, widely involved in the regulation of molecular pathways and trans‐differentiation of cell phenotypes.^8^, ^9^, ^10^, ^11^, ^12^, ^13^, ^14^ An increasing number of reports indicates that a group of miRs are involved in the differentiation of mesenchymal cells or myofibroblasts into osteoblastic phenotype and heterotopic ossification.^15^, ^16^, ^17^, ^18^, ^19^, ^20^, ^21^, ^22^, ^23^, ^24^, ^25^, ^26^ MiR‐204, miR‐29b, and miR‐30b can block the differentiation of mesenchymal cells into osteoblasts and are regarded as new target for early intervention of CAVD.^7^, ^27^, ^28^, ^29^ miR‐29b is reported to participate in the osteoblast differentiation of bone marrow stromal cells, osteogenic differentiation of somatic stem cells, and the calcification vascular smooth muscle cells through modulation of proteins such as BMPs, CDK6, and HDAC4 pathway.^30^, ^31^, ^32^ However, the role of miR‐29b in the CAVD has not been reported so far.

MiR‐29 family including miR‐29a, ‐29b, and ‐29c, is widely expressed in mature tissue cells, especially in the heart, lung, and kidney tissues.^33^, ^34^, ^35^, ^36^, ^37^ Among them, miR‐29b is a negative regulator of TGF‐β, located in the downstream of TGF‐β signaling pathway and the cross‐talk between miR‐29b, TGF‐β, and Smad constitutes an important regulator of extracellular matrix synthesis and is closely related to heart, lung, and kidney fibrosis processes.^35^, ^36^

Aortic stromal cells and osteoblasts are derived from mesenchymal cells but differ by the osteoblast‐specific expression of Cbfa1 and osteocalcin. Mesenchymal cells, aortic valve interstitial cells, and vascular smooth muscle cells present similarity of differentiation pathways. The calcification of aortic valve interstitial cells (VICs) is reported to be involved in the pathogenesis of CAVD. In addition, miR‐29b is involved in the osteoblastic differentiation of vascular smooth muscle cells and mesenchymal stem cells.^30^, ^31^, ^33^, ^37^ Thus, we hypothesized that miR‐29b could be involved in the osteoblast differentiation of hAVICs and could be an important regulator of heterotopic ossification of the aortic valve.

Therefore, in the present study, we performed an in vitro study to test our hypothesis that miR‐29b promotes the calcification of hAVICs. We found that miR‐29b markedly induces the osteoblastic differentiation and calcification of hAVICs by negatively regulating TGF‐β3 and activating wnt/β‐catenin and RUNX2/Smad3 signaling pathways.

## 2 MATERIALS AND METHODS

### 2.1 Ethics statement

Human valve studies were approved by the Institutional Review Board at Shanghai Zhoupu Hospital and conformed to the principles outlined in the Declaration of Helsinki. Written consent was obtained from all participants involved in this study.

### 2.2 Human aortic valve tissue

Human aortic valve tissue specimens were obtained from non‐syndromic adult patients at Shanghai Zhoupu Hospital who had calcific aortic valve disease and were undergoing aortic valve replacement (mean age  =  64, range 51–82 years), and from age‐matched patients at the time of autopsy, who died of non‐cardiac causes. Patients with a history of infective endocarditis or rheumatic heart disease were excluded. Samples were processed and analyzed by immunohistochemistry to confirm the extent of angiogenesis. Briefly, 5 μm sections of diseased (*n* = 6) and normal human aortic valves (*n*  =  4) were used for 30 min antigen retrieval in a pressure cooker using 0.01 M Citrate buffer (pH 6.0). Next, sections were blocked with 3% H_2_O_2_ for 15 min, and 5% normal goat serum in 0.05% PBST prior to incubation with the primary CD31 Antibody (Monoclonal, JC/70A) or VEGF Antibody (Monoclonal, VG1) both from Thermo Fisher Scientific (Waltham, MA) overnight at 4°C. Subsequently, PBST was used to wash sections followed by incubation with biotinylated secondary antibody before development with 3,3′‐diaminobenzidine (DAB Peroxidase Substrate Kit, Vector Lab, Burlingame, CA) and haematoxylin counter‐staining.

### 2.3 Isolation of hAVICs

The hAVICs were obtained from the human aortic valve leaflets as described by Zhang et al.^7^ Firstly, the endothelial layer of the ventricular and aortic aspects and nonleaflet tissues were carefully removed. Next, the leaflets were plunged for 5 min in 0.25% trypsin at 37°C and subsequently cut into 3 × 3 mm pieces. The obtained pieces were further digested in trypsin for 2 h at 37°C. The ensued primary hAVICs were cultured in DMEM medium supplemented with l‐glutamine (0.584 g/L), 10% FBS, 10 U/L penicillin, and 10 μg/L streptomycin. Cells were passaged five times and examined for the expression of marker proteins and microscopically for purity before use in subsequent experiments.

### 2.4 Cell culture

The Fibroblast Cellutions Medium PLUS was used for the optimal growth and expansion of hAVICs. This medium is formulated (quantitatively and qualitatively) to provide a defined and optimally balanced nutritional environment that selectively promotes proliferation and growth of valvular interstitial cells in vitro. The cells were cultured at 37°C under 5% CO_2_ conditions, and the medium was replaced with fresh medium every 3 days.

### 2.5 Plasmid construction

The full‐length open reading frame of TGF‐β3 was cloned into pcDNA3.1 (+) to generate its expression vector. The wild‐type TGF‐β3 3′‐UTR (WT) was cloned into the pGL3‐basic vector (Promega, Madison, WI). The miR‐29b seed sequence in the 3′‐UTR site‐directed mutagenesis was performed to generate the mutated TGF‐β3 3′‐UTR (Mut) using the QuikChange™ Site‐Directed Mutagenesis Kit (Stratagene, La Jolla, CA).

### 2.6 Transient transfection

Monolayers of hAVICs (5 × 10^4^ cells/cm^2^) were transfected with 2 nM of the miRIDIAN miR‐29b mimic, miR‐29b inhibitor or scrambled controls (Thermo Fisher Scientific, Waltham, MA) or transfected with plasmids using the Dharmafect transfection reagent (Dharmacon, Lafayette, CO), according to the manufacturer's recommendations. The transfected cells were cultured for 21 days in the low serum medium (1% fetal bovine serum supplemented). The medium was changed every 3 days. The cells were harvested for subsequent analysis.

### 2.7 Luciferase assays

For luciferase assays, hAVICs were cultured in 24‐well plates and co‐transfected with luciferase reporter plasmid and miR‐29b mimics, inhibitor or their respective controls, and pRL‐TK vector (Promega, Madison, WI) using Lipofectamine 2000 (Invitrogen, Carlsbad, CA). After transfection, cells were gathered 48 h later and lysed prior to the measurement of luciferase activity with the Dual‐Luciferase Reporter Assay System (Promega). Renilla‐luciferase was used for normalization. The experiments were performed independently in triplicate.

### 2.8 Osteoblastic differentiation

Osteoblastic differentiation was induced by culturing cells in Fibroblast Cellutions Medium PLUS supplemented with 50 mg/mL ascorbate‐2‐phosphate and 10 mM β‐glycerol phosphate. Fibroblast Cellutions Medium PLUS was used as culture control. hAVICs transfected with miR‐29b mimic, inhibitor, or controls were cultured in the same conditions. After 21 days culture, cultured hAVICs were collected and submitted to the subsequent experiments.

### 2.9 Alizarin red S staining

The extent of mineralization of hAVICs was evaluated using the Alizarin red S staining. Primary hAVICs cultured in Fibroblast Cellutions Medium PLUS supplemented with 10 mM β‐GP were fixed in 70% ethanol for 1 h at room temperature and stained with 40 mM Alizarin red S for 10 min. Then, cells were washed with PBS to eliminate nonspecific staining and the stained matrix was photographed using a digital microscope. For quantification of staining, the Alizarin red S stain was released from the cell matrix by incubation in cetyl‐pyridinium chloride for 15 min and the amount of released dye was measured by spectrophotometry at 540 nm. The results were normalized to total cellular protein content.

### 2.10 Measurement of intracellular calcium content

Cell cultures were collected and decalcified in a solution of 0.6 N HCl for 24 h. Following decalcification, washing of cells with PBS, and solubilization with 0.1 N NaOH‐0.1% SDS, the content in calcium of the HCl supernatant was evaluated using the O‐Cresolphthalein Complexone (Sigma–Aldrich, St. Louis, MO) method. The cell number was normalized by protein amount of HAVICs.

### 2.11 Alkaline phosphatase (ALP) assay

ALP activity of HAVICs was detected using the Alkaline Phosphatase Activity Colorimetric Assay Kit (BioVision Inc., Mountain View, CA) following the manufacturer's protocol. Briefly, cultured cells were washed with PBS and homogenized in 50 μl Assay Buffer. Following, the lysates were centrifuged at 13 000*g* for 3 min to remove insoluble material and distributed into 96‐well plate at different concentration in equal volumes. Next, 50 μl of the 5 mM pNPP solution was added to each well containing the background controls and test samples, mixed and incubated for 60 min at 25°C in dark. The calculation of ALP activity was done after the establishment of a standard curve using the formula: ALP activity (U/mL) = A/V/T wherein A equals to the amount of pNP generated by each sample (in μmol), V the volume of sample in the well (in mL), and T the reaction duration (in minutes).

### 2.12 Immunofluorescence

Cells in the samples were collected and washed twice in PBS before being fixed in 2 mL 4% paraformaldehyde in laminar hood for 15 min. After that, the samples were washed twice in PBS and permeabilized by incubation with 2 mL of 0.1% Triton X‐100 in PBS for 15 min. Then, after washing thrice with PBS and blocking for 1 h, diluted primary antibodies were added for an overnight incubation at 4°C in dark. On the second day, after washing five times with PBS (5 min each), samples were incubated with the Alexa Fluor 488‐conjugated F(ab′) fragment of goat anti‐mouse IgG (H+L) secondary antibodies (dilution 1:400; Thermo Fisher Scientific) in a dark humidity chamber at 4°C for 1 h and subsequently washed six times with PBS. Finally, the samples were mounted with a mounting medium and cells were observed under an epifluorescence microscope (IX51, DP70 digital camera; Olympus, Tokyo, Japan) or under a confocal microscope (TCS SP2; Leica Microsystems, Wetzlar, Germany).

### 2.13 Real‐time quantitative PCR

Total RNA was isolated from the cultured hAVICs using Trizol chloroform method reagent according to the manufacturer's instructions (Invitrogen) and reverse transcribed into cDNA with a Toyoba reverse transcription kit (Fermentas, Canada). The RT‐qPCR was carried out using the ABI PRISM 7900 sequence detector system (Applied Biosystems, Foster City, CA) following the instructions recommended by the supplier. The *β‐actin* gene and U6 were used as endogenous control for gene and miR‐29b, respectively. PCR reaction mixture contained the SYBR Green/Fluorescein QPCR Master Mix (2X) (Fermentas, Canada), cDNA, and the age‐miR‐29b LNA™ PCR primer set (UniRT) for miR‐29b. The sequences of primers for real‐time quantitative PCR were as presented in Table 1. Relative gene expression level was calculated using the comparative Ct method formula 2^−ΔΔCt^. Three independent experiments were carried out in triplicate.

**Table 1:** Sequences(5′‐3′) of primers and probes. R represents “reverse” while F symbolizes “forward”

Gene	Sequences (5′‐3′) of primers
Hsa‐mir‐U6‐F	CTCGCTTCGGCAGCACA
Hsa‐mir‐U6‐R	AACGCTTCACGAATTTGCGT
Mir‐29b	TAGCACCATTTGAAATCAGTGTT
Mir‐21	TAGCTTATCAGACTGATGTTGA
Mir‐133a	TTTGGTCCCCTTCAACCAGCTG
Mir‐141	TAACACTGTCTGGTAAAGATGG
Mir‐135a	TATAGGGATTGGAGCCGTGGCG
Has‐mir‐206	TGGAATGTAAGGAAGTGTGTGG
GAPDH‐F	GATGCCCCCATGTTCGTCAT
GAPDH‐R	TCTTCTGGGTGGCAGTGATG
TGF‐β3‐F	ACTTGCACCACCTTGGACTTC
TGF‐β3‐R	GGTCATCACCGTTGGCTCA
Smad3‐F	CTTGGACCTGCAGCCAGTTA
Smad3‐R	TCCACTGCTGCATTCCTGTT
Wnt3‐F	AGGGCACCTCCACCATTTG
Wnt3‐R	GACACTAACACGCCGAAGTCA
beta‐catenin‐F	CATCTACACAGTTTGATGCTGCT
beta‐catenin‐R	GCAGTTTTGTCAGTTCAGGGA

### 2.14 Western blotting analysis

Total protein was extracted from cultured hAVICs using the radio immunoprecipitation assay (RIPA) buffer protein concentration in lysates measured with the BCA protein quantification kit. Then, 50 μg of protein per sample was separated at 100 V by loading onto 12% SDS‐polyacrylamide gels (SDS‐PAGE) and electro‐transferred to PVDF membrane at 200 mA. Next, after blocking in 5% non‐fat milk in 0.1 M PBS for 2 h at ambient temperature, membranes were incubated with primary antibodies against TGF‐β3, Wnt3, β‐catenin, type I collagen, osteopontin, ALP, and osteocalcin overnight in dark at 4°C. subsequently, the membranes were washed thrice prior to incubation with HRP conjugated rabbit‐anti‐goat secondary antibodies (1:40000) for 2 h at room temperature. Finally, the membranes were washed washing thrice in PBS and incubated 5 min with ECL reagent for revelation. The densitometry analyses were performed using the Image J software and protein relative levels were calculated using GAPDH as loading control.

### 2.15 Statistical analysis

The results were expressed as mean ± SD. The significance of differences was estimated by one‐way or two‐way ANOVA followed by Dunnett's multiple comparisons test. *P *< 0.05 was considered for statistical significance. All statistical analyses were performed using GraphPad Prism software for Windows.

## 3 RESULTS

### 3.1 hAVICs are susceptible to osteoblastic differentiation

**Figure 1:**
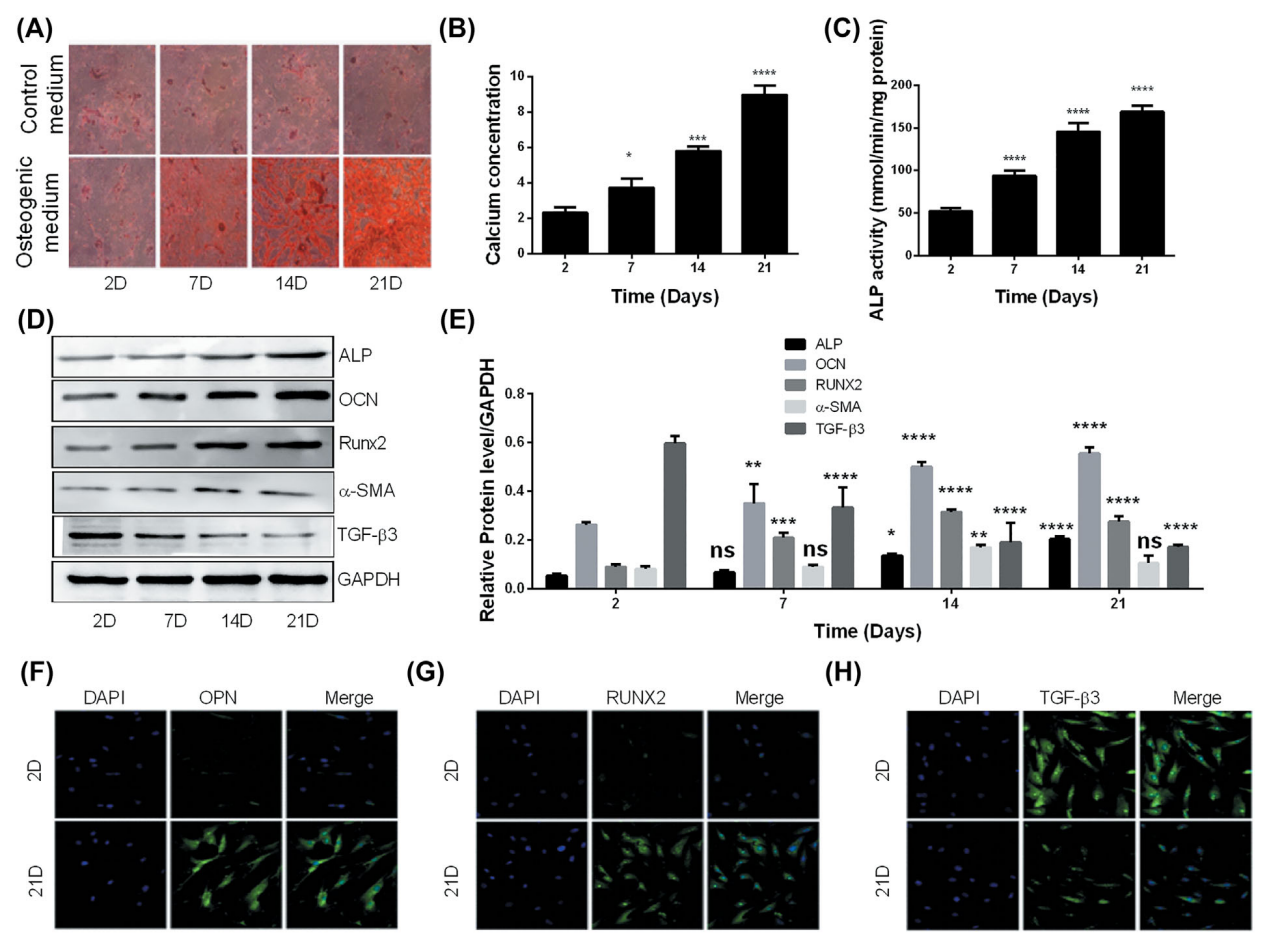
hAVICs are susceptible to Osteoblastic differentiation. A, Alazarin red S staining of hAVICs cultured in osteogenic or control medium for 21 days (representative microscopic images are shown, *n* = 3). B, Determination of calcium concentration in hAVICs cultured in osteogenic or control medium for 21 days. C, Determination of ALP activity in hAVICs cultured in osteogenic or control medium for 21 days (*n* = 3). D, Western blot analysis of osteogenic markers in hAVICs cultured in osteogenic or control medium for 21 days (representative microscopic images are shown, *n* = 3). E, Densitometry analysis of bands obtained from Western blot analysis of osteogenic markers in hAVICs cultured in osteogenic or control medium for 21 days (*n* = 3). F, immunofluorescence analysis of osteopontin (OPN) in hAVICs cultured in osteogenic or control medium for 21 days (*n* = 3). G, Immunofluorescence analysis of RUNX2 in hAVICs cultured in osteogenic or control medium for 21 days (*n* = 3). H, immunofluorescence analysis of TGF‐β3 in hAVICs cultured in osteogenic or control medium for 21 days (*n* = 3). The data are expressed as means ± SD, *n* = 3. *P < 0.05, **P < 0.01, ***P < 0.001, ****P < 0.0001 by comparison with the control cells (2 days)

Alazarin red S staining (Figure 1A) indicated that calcium deposits were not detected in hAVICs cultured in control medium, while positively‐stained particles were significantly and time‐dependently increased in hAVICs cultured in osteogenic medium. These observations were further confirmed by the quantitative measurement of the intracellular calcium content (Figure 1B) which was dramatically increased in hAVICs cultured in osteogenic medium compared to the control (*P* < 0.0001). Equally, ALP activity was significantly increased by osteogenic stimulation in a time‐dependent manner compared to hAVICs cultured in control medium (Figure 1C). The determination of protein levels using Western blotting (Figures 1D and 1E) and immunofluorescence (Figure 1F‐H) approaches showed that markers of osteogenic conversion, namely ALP, Osteocalcin, osteopontin, α‐SMA, and Runx2 were significantly increased in hAVICs cultured in osteogenic medium when compared with cells in the control group (*P* < 0.0001). On the contrary, the expression of the chondrogenic protein TGF‐β3 was significantly decreased. All these results indicated the osteoblastic differentiation and calcification of hAVICs and that the in vitro calcification model was established for subsequent studies.

### 3.2 miR‐29b is upregulated during the osteogenic differentiation

**Figure 2:**
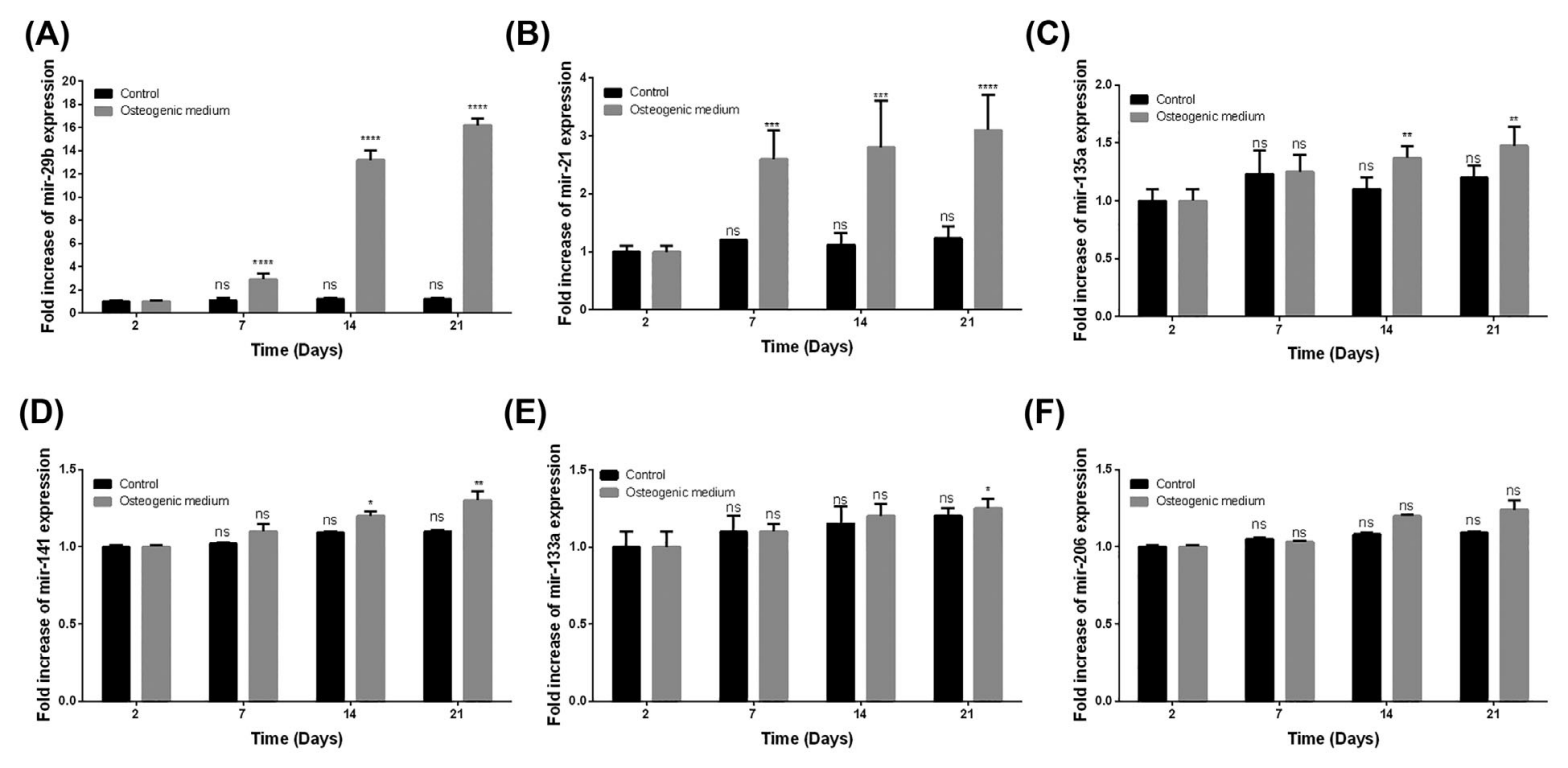
miR‐29b is upregulated during the osteogenic differentiation. The expressions of (A) miR‐29b, (B) miR‐21, (C) miR‐135a, (D) miR‐141, (E) miR‐133a, and (F) miR‐206 in cells cultured in osteogenic or control medium were measured using real time PCR at different time points. miR‐29b was the most upregulated miR during the osteogenic differentiation. The data are expressed as means ± SD, *n* = 3. ns = no significance, *P < 0.05, **P < 0.01, ***P < 0.001, ****P < 0.0001 by comparison with the control cells (2 days) and cells cultured in normal medium

In order to investigate miR‐29b involvement in the osteoblastic differentiation and calcification of hAVICs, the real‐time PCR method was used to detect the expression of miR‐29b, miR‐21, miR‐133a, miR‐144, miR‐135a, and miR‐206. After 7, 14, and 21 days of cell culture in the osteogenic medium, the expression of miR‐29b was significantly increased when compared with cells seeded in the normal medium (Figure 2A). The expression of miR‐21 was equally significantly increased from the 7th day, but its expression level was lower compared to miR‐29b (Figure 2B). The expressions of miR‐135a and miR‐141 became significantly increased from the 14th day, but their expression levels were also lower compared to miR‐29b (Figures 2C and 2D). The expression of miR‐133a became significantly increased at day 21 while no significant change was observed between cells cultured in osteogenic medium and control medium regarding the expression of miR‐206 (Figures 2E and 2F). These results suggested a potential robust implication of mir‐29b in the osteoblastic differentiation and calcification of hAVICs.

### 3.3 Inhibition of miR‐29b alleviates the osteoblastic differentiation and calcification of hAVICs

**Figure 3:**
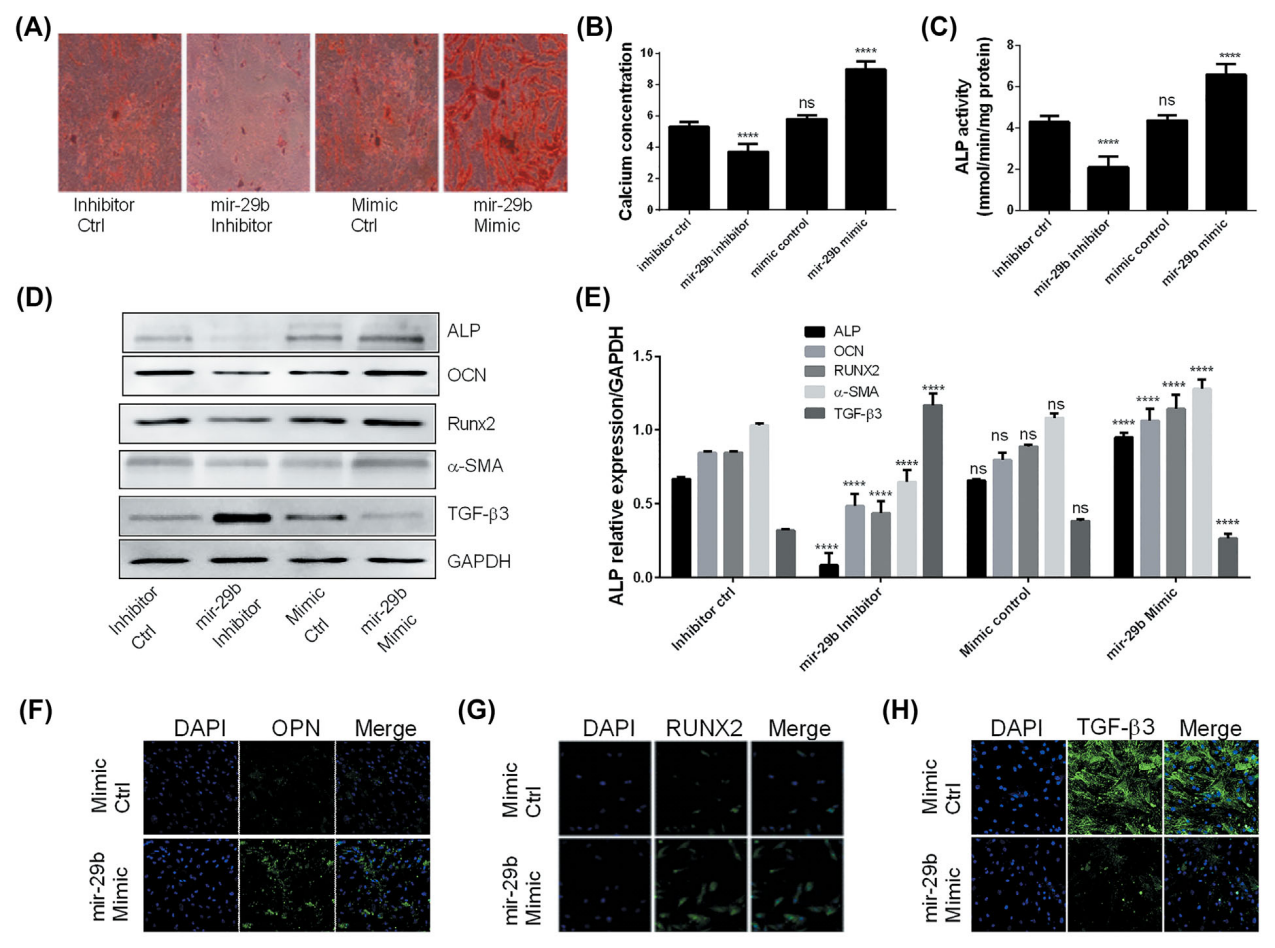
Inhibition of miR‐29b alleviates the osteoblastic differentiation and calcification of hAVICs. A, Alazarin red S staining of hAVICs transfected with miR‐29b mimics, inhibitor or negative controls, and cultured in osteogenic or control medium for 21 days (representative microscopic images are shown, *n* = 3). B, Determination of calcium concentration in hAVICs transfected with miR‐29b mimics, inhibitor or negative controls, and cultured in osteogenic or control medium for 21 days (*n* = 3). C, Determination of ALP activity in hAVICs transfected with miR‐29b mimics, inhibitor or negative controls, and cultured in osteogenic or control medium for 21 days (representative microscopic images are shown, *n* = 3). D, Western blot analysis of osteogenic markers in hAVICs transfected with miR‐29b mimics, inhibitor or negative controls, and cultured in osteogenic or control medium for 21 days (representative microscopic images are shown, *n* = 3). E, Densitometry analysis of bands obtained from Western blot analysis of osteogenic markers in hAVICs transfected with miR‐29b mimics, inhibitor of negative controls, and cultured in osteogenic or control medium for 21 days (*n* = 3). F, Immunofluorescence analysis of osteopontin (OPN) in hAVICs transfected with miR‐29b mimics or negative controls and cultured in osteogenic or control medium for 21 days (*n* = 3). G, Immunofluorescence analysis of RUNX2 in hAVICs transfected with miR‐29b mimics or negative control and cultured in osteogenic or control medium for 21 days (*n* = 3). H, Immunofluorescence analysis of TGF‐β3 in hAVICs transfected with miR‐29b mimics or negative controls and cultured in osteogenic or control medium for 21 days (*n* = 3). The data are expressed as means ± SD, *n* = 3, ****P < 0.0001 by comparison with the control cells (inhibitor control or mimic control), ns = no significance

To determine the effect of miR‐29b on the osteoblastic differentiation and induction of calcification, hAVICs were transfected with either mir‐29b mimics, mir‐29b inhibitor, or their respective controls and cultured in the control or osteogenic medium. The osteogenic medium triggered the osteoblastic differentiation of cells transfected with control oligonucleotides (Figure 3A). In hAVICs transfected with mir‐29b mimics, the osteoblastic differentiation was further promoted. However, this phenotype was significantly inhibited in hAVICs transfected with miR‐29b inhibitor (Figure 3A). These results were further confirmed by the results obtained from the measurement of calcium content which was also blocked by miR‐29b inhibition (Figure 3B). The enhanced ALP activity induced by osteogenic medium was repressed significantly with the miR‐29b inhibitor (Figure 3C). Equally, Western blotting (Figures 3D and 3E) and immufluorescence analyses (Figure 3F‐H) showed that the expressions of other osteogenic markers were equally downregulated following miR‐29b inhibition. On the inverse, TGF‐β3 was significantly upregulated after miR‐29b inhibition but downregulated by its mimics. The present results indicated that miR‐29b is a positive regulator of osteogenic differentiation of hAVICs

### 3.4 TGF‐β3 is a direct target of miR‐29b

**Figure 4:**
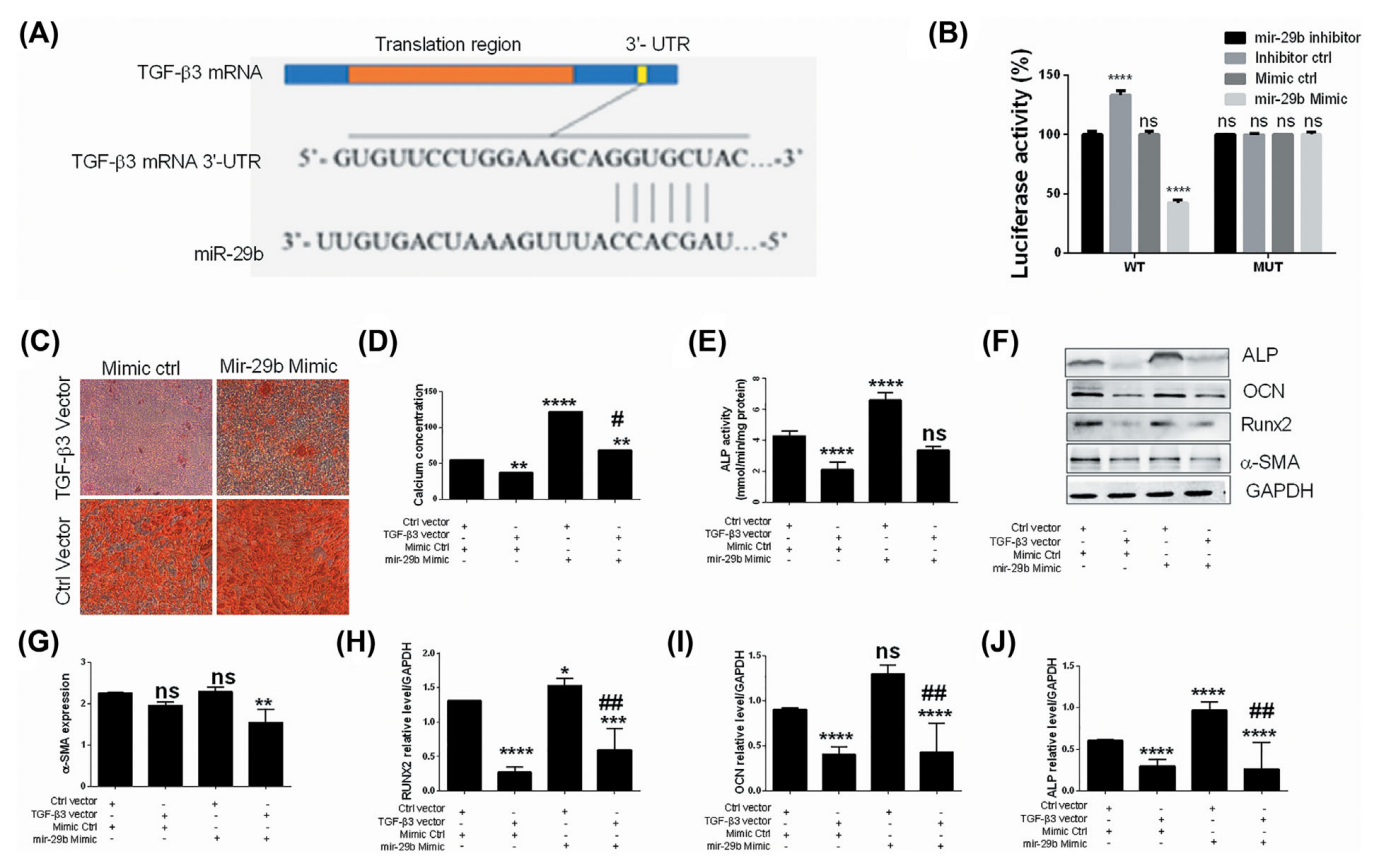
TGF‐β3 is a direct target of miR‐29b. A, Bioinformatics predicted TGF‐β3 mRNA 3′‐UTR as a direct target of miR‐29b. B, Luciferase test indicated TGF‐β3 mRNA 3′‐UTR as a direct target of miR‐29b (*n* = 3). C, Alazarin red S staining indicated that overexpression of TGF‐β3 abrogated the osteogenic activity of miR‐29b (representative microscopic images are shown, *n* = 3). D, Determination of calcium concentration indicated that overexpression of TGF‐β3 abrogated the miR‐29b‐induced calcium deposition (*n* = 3). E, Determination of ALP activity indicated that overexpression of TGF‐β3 abrogated the miR‐29b‐induced ALP activity (*n* = 3). F, Western blot analysis indicated that overexpression of TGF‐β3 abrogated the effect of miR‐29b on the expression of osteogenic markers (only representative bands are shown, *n* = 3). G,H, Densitometry analysis of bands obtained from Western blot analysis of osteogenic markers in hAVICs (*n* = 3). The data are expressed as means ± SD, *n* = 3, *P < 0.05, **P < 0.01, ***P < 0.001, ****P < 0.0001 by comparison with the control cells (control vector or mimic control), ns = no significance. #*P* < 0.05, ##*P* < 0.01 when compared to control mimics

In order to investigate the probable implication of TGF‐β3, Western blot analysis was performed and the results (Figures 1D and 1E) revealed that the osteogenic induction led to down‐regulation of TGF‐β3, suggesting its implication in the osteogenic differentiation. Using bioinformatics, we found that the mRNA 3′‐UTR region of TGF‐β3 was highly matched to the “seed sequence” of miR‐29b, suggesting that TGF‐β3 may be regulated by miR‐29b (Figure 4A). Further, luciferase reporter assay showed that miR‐29b inhibitor upregulated the expression of TGF‐β3 in cells cotransfected with the wild type 3′‐UTR of TGF‐β3 while in cells cotransfected with the mutated 3′‐UTR of TGF‐β3, no change was found (Figure 4B). Besides, miR‐29b mimics downregulated TGF‐β3 expression in cells cotransfected with the wild type 3′‐UTR of TGF‐β3 while in cells cotransfected with the mutated 3′‐UTR of TGF‐β3, no change was found (Figure 4B). These results suggested that miR‐29b may induce the osteoblastic differentiation of hAVICs by directly targeting TGF‐β3, postranscriptionally. To verify this hypothesis, TGF‐β3 and miR‐29b were co‐expressed in hAVICs which were subsequently cultured in osteogenic medium. The results showed that overexpression of TGF‐β3 significantly and partially reversed the osteogenic effect of miR‐29b as indicated by alizarin red S staining (Figure 4C), the determination of calcium concentration (Figure 4D) and ALP activity (Figure 4E). Western blot analysis further indicated that TGF‐β3 overexpression significantly reversed the miR‐29b‐induced Runx2 and OCN expressions and ALP activity (Figure 4F‐J). These results indicated that TGF‐β3 is a direct target of miR‐29b, suggesting that downregulation of TGF‐β3 by miR‐29b is involved in the osteoblastic differentiation of hAVICs.

### 3.5 miR‐29b downregulates TGF‐β3 via activation of wnt3/β‐catenin/Smad3 signaling pathways

**Figure 5:**
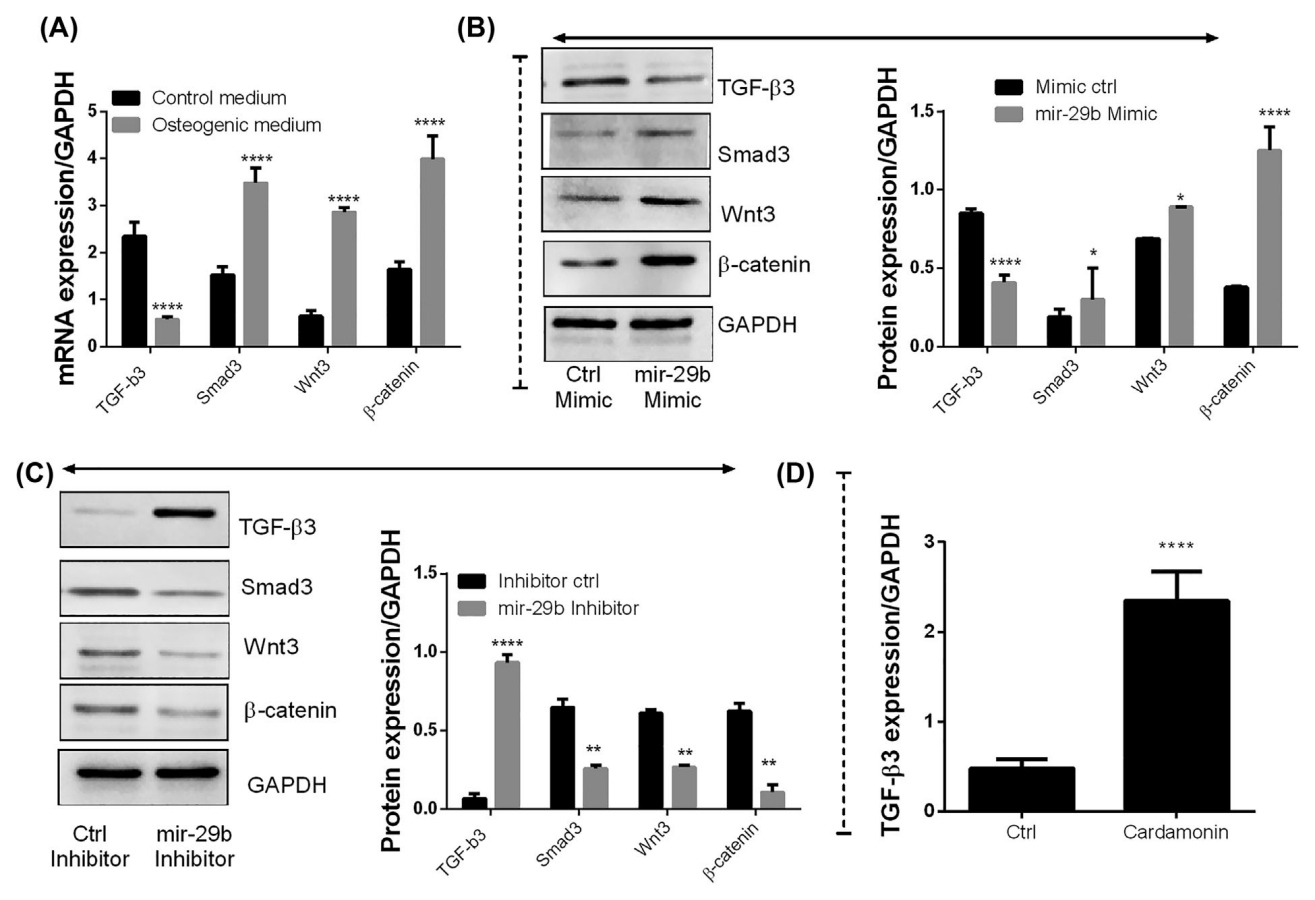
miR‐29b downregulates TGF‐β3 via activation of wnt3/β‐catenin/smad3 signaling pathways. A, mRNA expressions of *TGF‐β3, wnt3, β‐catenin*, and *smad3* in hAVICs cultured in osteogenic or control medium were determined using the quantitative real time PCR (*n* = 3). B, Protein expressions of TGF‐β3, wnt3, β‐catenin, and smad3 in hAVICs transfected with miR‐29b mimics or negative control were determined using Western blotting (*n* = 3). C, Protein expressions of TGF‐β3, wnt3, β‐catenin, and smad3 in hAVICs transfected with miR‐29b inhibitor or negative control were determined using Western blotting (*n* = 3). D, Inhibition of wnt3/β‐catenin pathway with Cardamonin induced the expression of TGF‐β3 (*n* = 3). The data are expressed as means ± SD, *n* = 3, *P < 0.05, **P < 0.01, ****P < 0.0001 by comparison with mimic control, inhibitor control, control medium, or Cardamonin‐untreated cells

The hAVICs were cultured in different media as described in the methods. The mRNA expression of *TGF‐β3, Smad3, wnt3*, and *β‐catenin* in hAVICs were measured by real‐time RT‐PCR. As shown in Figure 5A, *Smad3, wnt3, and β‐catenin* were significantly increased whereas *TGF‐β3* was significantly down‐regulated in hAVICs cultured in osteogenic medium compared to the control at the mRNA level (*P < 0.05 vs control), suggesting that TGF‐β3 and wnt3/β‐catenin/Smad3 signaling pathways are involved in the osteogenic differentiation of hAVICs.

To determine the effects of miR‐29b on the expression of these pathways, hAVICs were transfected with mir‐29b scramble or mimics and cultured in osteogenic media. Our data showed that inhibition of mir‐29b in hAVICs was followed by increased expression of Smad3, wnt3, and β‐catenin while TGF‐β3 was significantly down‐regulated when compared to scramble group cultured in osteogenic medium at the protein levels. The inverse actions were found with hAVICs transfected with the mir‐29b inhibitor. Furthermore, the inhibition of wnt3/β‐catenin pathway with Cardamonin was followed by increased expression of TGF‐β3. These results indicated that mir‐29b promoted the osteogenic differentiation and calcification of hAVICS by inhibiting TGF‐β3 via activating the wnt3/β‐catenin/Smad3 axis.

## 4 DISCUSSION

Calcified aortic valve disease is one of the principal causes of death and morbidity due to cardiovascular diseases, especially in the elderly, and calcification play a major role in the pathogenesis of this disease.^38^ Unfortunately, pharmacological treatments are not yet available for this disease due to lack of knowledge about the molecular and cellular mechanisms involved in its pathophysiology. Explanted human aortic valve leaflets exhibit evidence of bone‐like calcification, and aortic valve interstitial cells (AVICs), as the principal cellular components of aortic valve tissue, are involved in the pathological process.^38^ Therefore, elucidation of the mechanisms underlying the process of valvular calcification and identification of the factors involved is important.

In the present study, we revealed that culturing hAVICs in osteogenic medium resulted in the osteoblastic transformation of these cells. This observation conformed with previous findings that conveyed the pro‐osteogenic response of AVICs isolated from stenotic valves.^6^, ^7^, ^38^, ^39^

In addition, we revealed that miR‐29b was highly expressed in calcific aortic leaflets and primary hAVICs cultured in osteogenic medium and that transfection of miRNA‐29b inhibitor into these cells blocks the osteoblastic transformation and the associated calcification process. To the best of our knowledge, this is the first report of miR‐29b upregulation in the osteoblast transformation of hAVICs. However, similar results have been reported for the mesenchymal stem cells, vascular smooth muscle cells, and somatic stem cells from human cord blood.^15^, ^30^, ^37^ On the contrary, miR‐29b was found to be lowly expressed during osteoblast differentiation of bone marrow stromal cells from Osteogenesis Imperfecta patients.^31^ These observations indicate that miR‐29b could play an important function in the osteogenesis and calcification of hAVICs, which needs further clarification.

Although several miRs that are expressed in hAVICs have been identified, investigation on their roles in valvular calcification is limited. Previous reports indicate that miR‐29 is involved in the osteoblastic differentiation of vascular smooth muscle cells by down‐regulating elastin expression.^37^ However, it is unknown if miR‐29b exerts any effect on calcification of hAVICs. In the present study, we showed that overexpression of miR‐29b increased ALP activity, osteocalcin secretion and the expression of Runx2 and induced the mineralization of hAVICs whereas inhibition of miR‐29b expression led to the opposite effects. This correlation between miR‐29b expression and the activity and levels of important markers of osteoblast differentiation demonstrated the role of miR‐29b as a positive regulator of osteogenic differentiation of hAVICs and that inhibition of this miR will be advantageous for the treatment of cardiovascular diseases, particularly CAVD.

To further investigate the molecular mechanism by which miR‐29b inhibits osteogenic differentiation, we conducted computational search for the target genes of miR‐29b and found that TGF‐β3 was a possible target because complementary sequence of miR‐29b was identified in the 3′‐UTR of its mRNA. This in silico result was further confirmed by luciferase assay and mechanistic studies indicated that miRNA‐29b acts as a positive regulator of osteoblast differentiation via suppressing TGF‐β3 and activating the wnt3, β‐catenin, Runx2, and Smad3 expressions.

TGF‐β3 is an essential prochondrogenesis transcription factor.^40^, ^41^, ^42^, ^43^ Our study revealed that TGF‐β3 was downregulated during osteogenesis and calcification of HAVICs. Smad3/RUNX2 and Wnt/β‐cantenin are important pathways of osteogenic differentiation.^44^, ^45^, ^46^ Furthermore, TGF‐β3 activated Smads interact with Runx2 to enhance the transcription of osteoblast‐specific genes, and the interaction of TGF‐β3 with Runx2 on the promoter of target genes controls osteoblast gene expression and differentiation. The regulation of Runx2 and Smad3 by different miRs in relation to osteogenic differentiation has been shown in previous studies.^47^, ^48^, ^49^ In the present study, we found that Runx2 or Smad3 were positively regulated by miR‐29b. These results confirm that miR‐29b promotes osteogenic differentiation by positively regulating Runx2 and Smad3. Similarly, mir‐29b upregulated the wnt3/β‐catenin signaling pathway.

## 5 CONCLUSION

The present study showed that miR‐29b acts as a positive regulator of osteogenic differentiation of HAVICs at least in part by targeting TGF‐β3/Smad3 and wnt3/β‐catenin signaling pathways and confirmed the involvement of miRs in valvular calcification. Our results indicate that miR‐29b could be a useful marker of osteogenic differentiation and the modulation of its expression could be a potential therapeutic strategy for the prevention or treatment of a variety of diseases associated with valvular calcification.

## CONFLICTS OF INTEREST

No competing financial interests exist.

## References

[1] Galeone A , Paparella D , Colucci S , Grano M , Brunetti G . The role of TNF‐alpha and TNF superfamily members in the pathogenesis of calcific aortic valvular disease. ScientificWorldJournal. 2013; 2013:875363. 24307884 10.1155/2013/875363 PMC3836568

[2] Ossareh S . Cardiac valvular calcification in hemodialysis patients. Iran J Kidney Dis. 2013; 7:77–79. 23485529

[3] Sanchez‐Perales C , Vazquez Ruiz de Castroviejo E , Garcia‐Cortes MJ , et al. Valvular calcifications at the start of dialysis predict the onset of cardiovascular events in the course of follow‐up. Nefrologia. 2015; 35:157–163. 26300509 10.1016/j.nefro.2015.05.017

[4] Thanassoulis G , Campbell CY , Owens DS , et al. Genetic associations with valvular calcification and aortic stenosis. N Engl J Med. 2013; 368:503–512. 23388002 10.1056/NEJMoa1109034 PMC3766627

[5] Towler DA . Molecular and cellular aspects of calcific aortic valve disease. Circ Res. 2013; 113:198–208. 23833294 10.1161/CIRCRESAHA.113.300155 PMC4057916

[6] Wirrig EE , Gomez MV , Hinton RB , Yutzey KE . COX2 inhibition reduces aortic valve calcification in vivo. Arterioscler Thromb Vasc Biol. 2015; 35:938–947. 25722432 10.1161/ATVBAHA.114.305159 PMC4979542

[7] Zhang M , Liu X , Zhang X , et al. MicroRNA‐30b is a multifunctional regulator of aortic valve interstitial cells. J Thorac Cardiovasc Surg. 2014; 147:e2. 10.1016/j.jtcvs.2013.05.011 23968872

[8] Aghagolzadeh P , Radpour R . New trends in molecular and cellular biomarker discovery for colorectal cancer. World J Gastroenterol. 2016; 22:5678–5693. 27433083 10.3748/wjg.v22.i25.5678 PMC4932205

[9] Catalanotto C , Cogoni C , Zardo G . MicroRNA in control of gene expression: an overview of nuclear functions. Int J Mol Sci. 2016; 17:1712. 10.3390/ijms17101712 PMC5085744 27754357

[10] Gareri C , De Rosa S , Indolfi C . MicroRNAs for restenosis and thrombosis after vascular injury. Circ Res. 2016; 118:1170–1184. 27034278 10.1161/CIRCRESAHA.115.308237

[11] Horak M , Novak J , Bienertova‐Vasku J . Muscle‐specific microRNAs in skeletal muscle development. Dev Biol. 2016; 410:1–13. 26708096 10.1016/j.ydbio.2015.12.013

[12] Kacperska MJ , Walenczak J , Tomasik B . Plasmatic microRNA as potential biomarkers of multiple sclerosis: literature review. Adv Clin Exp Med. 2016; 25:775–779. 27629854 10.17219/acem/60098

[13] Mari‐Alexandre J , Sanchez‐Izquierdo D , Gilabert‐Estelles J , Barcelo‐Molina M , Braza‐Boils A , Sandoval J . MiRNAs regulation and its role as biomarkers in endometriosis. Int J Mol Sci. 2016; 17:93. 10.3390/ijms17010093 PMC4730335 26771608

[14] Wang J , Liew OW , Richards AM , Chen YT . Overview of microRNAs in cardiac hypertrophy, fbrosis, and apoptosis. Int J Mol Sci. 2016; 17:749. 10.3390/ijms17050749 PMC4881570 27213331

[15] Eguchi T , Watanabe K , Hara ES , Ono M , Kuboki T , Calderwood SK . OstemiR: a novel panel of microRNA biomarkers in osteoblastic and osteocytic differentiation from mesencymal stem cells. PLoS ONE. 2013; 8:e58796. 23533592 10.1371/journal.pone.0058796 PMC3606401

[16] Chen C , Cheng P , Xie H , et al. MiR‐503 regulates osteoclastogenesis via targeting RANK. J Bone Miner Res. 2014; 29:338–347. 23821519 10.1002/jbmr.2032

[17] Ji X , Chen X , Yu X . MicroRNAs in osteoclastogenesis and function: potential therapeutic targets for osteoporosis. Int J Mol Sci. 2016; 17:349. 27005616 10.3390/ijms17030349 PMC4813210

[18] Meng YB , Li X , Li ZY , et al. MicroRNA‐21 promotes osteogenic differentiation of mesenchymal stem cells by the PI3K/beta‐catenin pathway. J Orthop Res. 2015; 33:957–964. 25728838 10.1002/jor.22884

[19] Song Q , Zhong L , Chen C , et al. MiR‐21 synergizes with BMP9 in osteogenic differentiation by activating the BMP9/Smad signaling pathway in murine multilineage cells. Int J Mol Med. 2015; 36:1497–1506. 26460584 10.3892/ijmm.2015.2363 PMC4678163

[20] Sun Y , Xu L , Huang S , et al. Mir‐21 overexpressing mesenchymal stem cells accelerate fracture healing in a rat closed femur fracture model. Biomed Res Int. 2015; 2015:412327. 25879024 10.1155/2015/412327 PMC4386680

[21] Tang P , Xiong Q , Ge W , Zhang L . The role of microRNAs in osteoclasts and osteoporosis. RNA Biol. 2014; 11:1355–1363. 25692234 10.1080/15476286.2014.996462 PMC4615571

[22] Trohatou O , Zagoura D , Bitsika V , et al. Sox2 suppression by miR‐21 governs human mesenchymal stem cell properties. Stem Cells Transl Med. 2014; 3:54–68. 24307698 10.5966/sctm.2013-0081 PMC3902287

[23] Wang Z , Wu G , Feng Z , et al. Microarc‐oxidized titanium surfaces functionalized with microRNA‐21‐loaded chitosan/hyaluronic acid nanoparticles promote the osteogenic differentiation of human bone marrow mesenchymal stem cells. Int J Nanomedicine. 2015; 10:6675–6687. 26604744 10.2147/IJN.S94689 PMC4630184

[24] Wang Z , Wu G , Wei M , et al. Improving the osteogenesis of human bone marrow mesenchymal stem cell sheets by microRNA‐21‐loaded chitosan/hyaluronic acid nanoparticles via reverse transfection. Int J Nanomedicine. 2016; 11:2091–2105. 27274237 10.2147/IJN.S104851 PMC4876805

[25] Yang N , Wang G , Hu C , et al. Tumor necrosis factor alpha suppresses the mesenchymal stem cell osteogenesis promoter miR‐21 in estrogen deficiency‐induced osteoporosis. J Bone Miner Res. 2013; 28:559–573. 23074166 10.1002/jbmr.1798

[26] Wei F , Liu D , Feng C , et al. MicroRNA‐21 mediates stretch‐induced osteogenic differentiation in human periodontal ligament stem cells. Stem Cells Dev. 2015; 24:312–319. 25203845 10.1089/scd.2014.0191 PMC4303015

[27] Cui RR , Li SJ , Liu LJ , et al. MicroRNA‐204 regulates vascular smooth muscle cell calcification in vitro and in vivo. Cardiovasc Res. 2012; 96:320–329. 22871591 10.1093/cvr/cvs258

[28] Panizo S , Naves‐Diaz M , Carrillo‐Lopez N , et al. MicroRNAs 29b, 133b, and 211 regulate vascular smooth muscle calcification mediated by high phosphorus. J Am Soc Nephrol. 2016; 27:824–834. 26187577 10.1681/ASN.2014050520 PMC4769184

[29] Wang Y , Chen S , Deng C , et al. MicroRNA‐204 targets runx2 to attenuate BMP‐2‐induced osteoblast differentiation of human aortic valve interstitial cells. J Cardiovasc Pharmacol. 2015; 66:63–71. 25806689 10.1097/FJC.0000000000000244

[30] Trompeter HI , Dreesen J , Hermann E , et al. MicroRNAs miR‐26a, miR‐26b, and miR‐29b accelerate osteogenic differentiation of unrestricted somatic stem cells from human cord blood. BMC Genomics. 2013; 14:111. 23418963 10.1186/1471-2164-14-111 PMC3637629

[31] Kaneto CM , Lima PS , Zanette DL , et al. COL1A1 and miR‐29b show lower expression levels during osteoblast differentiation of bone marrow stromal cells from Osteogenesis Imperfecta patients. BMC Med Genet. 2014; 15:45. 24767406 10.1186/1471-2350-15-45 PMC4101867

[32] Laxman N , Rubin CJ , Mallmin H , et al. Global miRNA expression and correlation with mRNA levels in primary human bone cells. RNA. 2015; 21:1433–1443. 26078267 10.1261/rna.049148.114 PMC4509933

[33] Bretschneider M , Busch B , Mueller D , et al. Activated mineralocorticoid receptor regulates micro‐RNA‐29b in vascular smooth muscle cells. FASEB J. 2016; 30:1610–1622. 26728178 10.1096/fj.15-271254

[34] Guo W , Benlhabib H , Mendelson CR . The microRNA 29 family promotes type II cell differentiation in developing lung. Mol Cell Biol. 2016; 36:2141. 27215389 10.1128/MCB.00096-16 PMC4968214

[35] Li J , Cen B , Chen S , He Y . MicroRNA‐29b inhibits TGF‐beta1‐induced fibrosis via regulation of the TGF‐beta1/Smad pathway in primary human endometrial stromal cells. Mol Med Rep. 2016; 13:4229–4237. 27035110 10.3892/mmr.2016.5062 PMC4838148

[36] Liang C , Bu S , Fan X . Suppressive effect of microRNA‐29b on hepatic stellate cell activation and its crosstalk with TGF‐beta1/Smad3. Cell Biochem Funct. 2016; 34:326–333. 27273381 10.1002/cbf.3193 PMC5089641

[37] Sudo R , Sato F , Azechi T , Wachi H . MiR‐29‐mediated elastin down‐regulation contributes to inorganic phosphorus‐induced osteoblastic differentiation in vascular smooth muscle cells. Genes Cells. 2015; 20:1077–1087. 26610870 10.1111/gtc.12311

[38] Zeng Q , Song R , Ao L , et al. Notch1 promotes the pro‐osteogenic response of human aortic valve interstitial cells via modulation of ERK1/2 and nuclear factor‐kappaB activation. Arterioscler Thromb Vasc Biol. 2013; 33:1580–1590. 23640488 10.1161/ATVBAHA.112.300912 PMC3778193

[39] White MP , Theodoris CV , Liu L , et al. NOTCH1 regulates matrix gla protein and calcification gene networks in human valve endothelium. J Mol Cell Cardiol. 2015; 84:13–23. 25871831 10.1016/j.yjmcc.2015.04.006 PMC4468000

[40] Dahlin RL , Ni M , Meretoja VV , Kasper FK , Mikos AG . TGF‐beta3‐induced chondrogenesis in co‐cultures of chondrocytes and mesenchymal stem cells on biodegradable scaffolds. Biomaterials. 2014; 35:123–132. 24125773 10.1016/j.biomaterials.2013.09.086 PMC3844679

[41] Lu CH , Lin KJ , Chiu HY , et al. Improved chondrogenesis and engineered cartilage formation from TGF‐beta3‐expressing adipose‐derived stem cells cultured in the rotating‐shaft bioreactor. Tissue Eng Part A. 2012; 18A:2114–2124. 10.1089/ten.TEA.2012.0010 22712565

[42] Zhang T , Wen F , Wu Y , et al. Cross‐talk between TGF‐beta/SMAD and integrin signaling pathways in regulating hypertrophy of mesenchymal stem cell chondrogenesis under deferral dynamic compression. Biomaterials. 2015; 38:72–85. 25453975 10.1016/j.biomaterials.2014.10.010

[43] Zheng D , Dan Y , Yang SH , et al. Controlled chondrogenesis from adipose‐derived stem cells by recombinant transforming growth factor‐beta3 fusion protein in peptide scaffolds. Acta Biomater. 2015; 11:191–203. 25257317 10.1016/j.actbio.2014.09.030

[44] Javed A , Bae JS , Afzal F , et al. Structural coupling of Smad and Runx2 for execution of the BMP2 osteogenic signal. J Biol Chem. 2008; 283:8412–8422. 18204048 10.1074/jbc.M705578200 PMC2417186

[45] Zhang JF , Li G , Chan CY , et al. Flavonoids of Herba Epimedii regulate osteogenesis of human mesenchymal stem cells through BMP and Wnt/beta‐catenin signaling pathway. Mol Cell Endocrinol. 2010; 314:70–74. 19703516 10.1016/j.mce.2009.08.012

[46] Kim KO , Sampson ER , Maynard RD , et al. Ski inhibits TGF‐beta/phospho‐Smad3 signaling and accelerates hypertrophic differentiation in chondrocytes. J Cell Biochem. 2012; 113:2156–2166. 22461172 10.1002/jcb.24089 PMC3324639

[47] Hassan MQ , Gordon JA , Beloti MM , et al. A network connecting Runx2, SATB2, and the miR‐23a∼27a∼24‐2 cluster regulates the osteoblast differentiation program. Proc Natl Acad Sci USA. 2010; 107:19879–19884. 20980664 10.1073/pnas.1007698107 PMC2993380

[48] Zhang Y , Xie RL , Croce CM , et al. A program of microRNAs controls osteogenic lineage progression by targeting transcription factor Runx2. Proc Natl Acad Sci USA. 2011; 108:9863–9868. 21628588 10.1073/pnas.1018493108 PMC3116419

[49] Cheung KS , Sposito N , Stumpf PS , Wilson DI , Sanchez‐Elsner T , Oreffo RO . MicroRNA‐146a regulates human foetal femur derived skeletal stem cell differentiation by down‐regulating SMAD2 and SMAD3. PLoS ONE. 2014; 9:e98063. 24892945 10.1371/journal.pone.0098063 PMC4043645

